# Clinical-Genetic Approach to Conditions with Macrocephaly and ASD/Behaviour Abnormalities: Variants in *PTEN* and *PPP2R5D* Are the Most Recurrent Gene Mutations in a Patient-Oriented Diagnostic Strategy

**DOI:** 10.3390/genes16040469

**Published:** 2025-04-20

**Authors:** Federica Francesca L’Erario, Annalisa Gazzellone, Ilaria Contaldo, Chiara Veredice, Marina Carapelle, Anna Gloria Renzi, Clarissa Modafferi, Marta Palucci, Pino D’Ambrosio, Elena Sonnini, Lorenzo Loberti, Arianna Panfili, Emanuela Lucci Cordisco, Pietro Chiurazzi, Valentina Trevisan, Chiara Leoni, Giuseppe Zampino, Maria Grazia Pomponi, Daniela Orteschi, Marcella Zollino, Giuseppe Marangi

**Affiliations:** 1 Unit of Medical Genetics, Section of Genomic Medicine, Department of Life Sciences and Public Health, Università Cattolica del Sacro Cuore, Fondazione Policlinico Universitario Agostino Gemelli IRCCS, 00168 Rome, Italy; federica.lerario01@icatt.it (F.F.L.); annalisa.gazzellone01@icatt.it (A.G.); marina.carapelle@unicatt.it (M.C.); annagloria.renzi@unicatt.it (A.G.R.); giuseppe.marangi@unicatt.it (G.M.); 2 Child Neurology and Psychiatry Unit, Department of Neuroscience, Università Cattolica del Sacro Cuore, Fondazione Policlinico Universitario Agostino Gemelli IRCCS, 00168 Rome, Italy; 3 Medical Genetics, University of Siena, 53100 Siena, Italy; 4 Scientific Directorate, Fondazione Policlinico Universitario Agostino Gemelli IRCCS, 00168 Roma, Italy; 5 Center for Rare Diseases and Birth Defects, Department of Woman and Child Health and Public Health, Fondazione Policlinico Universitario Agostino Gemelli IRCCS, 00168 Rome, Italy

**Keywords:** macrocephaly, neurodevelopmental disorders, autism, behaviour abnormalities, *PTEN*, *PPP2R5D*

## Abstract

Background: Macrocephaly can be a component manifestation of several monogenic conditions, in association with intellectual disability/developmental delay (ID/DD) behaviour abnormalities, including autism spectrum disorders (ASD), and variable additional features. On the other hand, idiopathic ASD can present with developmental delay and macrocephaly. Methods: We carried out a retrospective analysis of a cohort of 78 patients who were tested from February 2017 to December 2024 by high-throughput sequencing of a panel of 27 genes (*ABCC9*, *AKT1*, *AKT2*, *AKT3*, *BRWD3*, *DIS3L2*, *DNMT3A*, *EZH2*, *GPC3*, *GPC4*, *HERC1*, *MED12*, *MTOR*, *NFIA*, *NFIX*, *NSD1*, *PDGFRB*, *PIK3CA*, *PIK3R1*, *PIK3R2*, *PPP2R1A*, *PPP2R5D*, *PTEN*, *RAB39B*, *RNF135*, *SETD2,* and *TBC1D7*) because of neurodevelopmental impairment, including ID/DD, ASD/behaviour abnormalities associated with macrocephaly, mimicking to a large extent idiopathic ASD. Results: Pathogenic variants leading to the diagnosis of monogenic conditions were detected in 22 patients (28%), including *NSD1* (2), *PTEN* (16), and *PPP2R5D* (4). Distinctive of the *PTEN*-associated phenotype were true macrocephaly (100%), ASD or behaviour abnormalities (92%), mild/borderline ID (79%), and no facial dysmorphisms. Typical of the *PPP2R5D*-associated phenotype were relative macrocephaly (75%), a few unspecific peculiar facial characteristics (50%), and a more variable presentation of the neurodevelopmental phenotype. Conclusions: Pathogenic variants in *PTEN* and *PPP2R5D* are the most recurrent gene mutations in a patient-oriented procedure for the genetic diagnosis of apparently idiopathic ASD and behaviour abnormalities associated with macrocephaly. The clinical applicability of the presented diagnostic strategy is discussed.

## 1. Introduction

The development of high-throughput sequencing (HTS) methods has drastically transformed the landscape of genetic diagnosis in the field of neurodevelopmental disorders. The challenges of HTS are primarily related to the large number of variants that must be screened to identify those of interest, specifically those responsible for the observed phenotype in a diagnostic context. Therefore, a precise definition and characterisation of the clinical phenotype has a pivotal role to play in the prioritisation of variants with a higher likelihood of being responsible for the observed disorder.

Macrocephaly can be a clinical sign that guides the diagnostic process, especially in the setting of neurodevelopmental disorders (NDDs) and/or autism spectrum disorders (ASD) or related behaviour abnormalities. Macrocephaly is defined as a head circumference (HC) exceeding two standard deviations (SD) above the population mean for age and sex [1]. Macrocephaly can be benign, and in these cases the skull enlargement is associated neither with neurological abnormalities nor with developmental impairment. On the contrary, if macrocephaly occurs in association with additional features, especially with neurodevelopmental delay, it can be an important indicator of a variety of monogenic conditions [2]. True, or absolute, macrocephaly refers to a real increase in HC, resulting in values significantly higher, more than 2 SD, than the population means. Relative macrocephaly refers to a relatively mild degree of macrocephaly in which the head circumference is not above two SD from the mean but appears disproportionately large when other factors such as body stature are taken into account (HP:0004482). It may be due to a normal variation in growth or it may be a hereditary trait [3] common in some families [4].

In the field of paediatric genetics, the association of macrocephaly and autism is a well-established occurrence. Notable examples of this relationship include the macrocephaly/Autism Syndrome (MIM: 605309), which is linked to pathogenic variants (PVs) in the *PTEN* gene (MIM: 601728), and Malan syndrome (MIM: 614753), which is caused by PVs in the *NFIX* gene (MIM: 164005). In both cases, clinical manifestations can be limited to ASD and macrocephaly, with neurodevelopmental impairment that can be more often of mild entity or even absent. Notably, PVs in the *PTEN* gene in this clinical category have been reported to range from 1% to 22%, as reviewed by Kaymakcalan and coll [5]. Since *PTEN* acts as an oncogene, the achievement of the genetic diagnosis has great relevance for planning cancer surveillance. *PTEN*- and *NFIX*-associated disorders fall within monogenic conditions with well-established genotype–phenotype correlations and consistent nosology. However, idiopathic autism, which can be oligogenic/multifactorial in nature, can also be associated with macrocephaly and neurodevelopmental impairment as well [6].

This report is focused on a centre-specific experience with a cohort of paediatric patients who were referred because of ASD, or related behaviour abnormalities, and macrocephaly, associated with slight or even absent cognitive impairment, nearly normal motor milestones, and no true facial dysmorphisms. The aim of our study is to search for the frequency of monogenic conditions in individuals with apparently idiopathic ASD/behavioural abnormalities and macrocephaly.

## 2. Materials and Methods

### 2.1. Case Series

A total of 78 individuals were referred for genetic counselling by child neurologists from February 2017 to December 2024 because of a diagnosis of apparently idiopathic neurodevelopmental disorder, associated with relative or absolute macrocephaly. After performing a Chromosomal Microarray Analysis (CMA) by oligonucleotide-based array-CGH in 73, all patients with no genomic copy number variations were enrolled into targeted high-throughput sequencing (HTS) of the gene panel described here. Since a phenotype that is highly suggestive for a *PTEN* variant emerged at clinical evaluation in 5 patients, they underwent targeted HTS gene panel analysis first, with positive results, and array-CGH was not performed. MLPA analysis of the *PTEN*, *NSD1*, and *NFIX* genes was performed in a total of 50 patients, which obtained normal results on the CMA and gene analyses. In addition to a preliminary hypothesis of idiopathic neurodevelopmental disorders, the inclusion criteria were: speech delay/DD/ID; ASD/behaviour abnormalities; absolute or relative macrocephaly; absence of facial dysmorphisms, significant motor delay and hypotonia. The individual pattern of the neurodevelopmental impairment, including behaviour assessment, was established by child neurologists by using appropriated criteria according to patients’ age, including a variable association of the WAIS IV, WISC IV, GRIFFITH, LEITHER-3, VINELAND-II, and ADOS scales. The diagnosis of ASD was based on DSM-V criteria [7], or on the basis of a multidisciplinary evaluation, including a psychologist and developmental paediatrician.

Among the 78 patients, 22 were diagnosed with a pathogenic variant in *PTEN* (16), *PPP2R5D* (4), and *NSD1* (2).

Following institutional review board approval, we present the clinical and genetic data of these 22 patients, who were all retrospectively re-evaluated. They included 8 females and 14 males. The mean age at last observation was 7.3 years (range 1–19). For each subject, their personal and family medical history was recorded, along with their growth parameters (body weight, length/height, and head circumference) at birth and at the time of the last clinical evaluation. The presence of hypotonia, EEG abnormalities and/or seizures, motor and language delay, intellectual disability, behavioural issues and/or ASD, neuroimaging anomalies, distinctive facial features, and any other additional features was also re-evaluated.

For the definition of intellectual disability and to specify the degree of severity, we used the following categories, as provided in the Human Phenotype Ontology (HPO) [8], which defines borderline ID as an IQ in the range of 70–85, mild ID in the range of 50–69, moderate ID in the range of 35–49, and severe ID in the range of 20–34 [8].

Written informed consent to participate in this study and to report the case details was obtained from the patients or their parents on each occasion, with the exception of one subject with a de novo pathogenic variant in *PTEN*. The clinical data of this last patient are not included in this paper.

### 2.2. Array-CGH

Array-CGH analysis was performed on DNA from peripheral blood cells by means of the commercial Agilent 4 × 60 kit (Agilent Technologies, Santa Clara, CA, USA), following the manufacturer’s instructions, using ADM-2 algorithm for the data analysis with Agilent CytoGenomics v5.4 software.

### 2.3. MLPA

Multiplex Ligation-dependent Probe Amplification analysis (MLPA) was performed with the P225-E1 *PTEN* and P026-E2 Sotos (*NSD1* and *NFIX*) kits (MRC Holland, Amsterdam, The Netherlands). The analysis was carried out using standard procedures, according to the manufacturer’s instructions. Fragment separation was performed by capillary electrophoresis on a SeqStudio 8 Flex Genetic Analyzer (Thermo Fisher Scientific, Waltham, MA, USA) and the results were analysed using Coffalyser.net v.240129.1959 (MRC Holland, Amsterdam, The Netherlands).

### 2.4. HTS Panel

HTS of a panel of 27 genes associated with syndromic macrocephaly and/or overgrowth (*ABCC9*, *AKT1*, *AKT2*, *AKT3*, *BRWD3*, *DIS3L2*, *DNMT3A*, *EZH2*, *GPC3*, *GPC4*, *HERC1*, *MED12*, *MTOR*, *NFIA*, *NFIX*, *NSD1*, *PDGFRB*, *PIK3CA*, *PIK3R1*, *PIK3R2*, *PPP2R1A*, *PPP2R5D*, *PTEN*, *RAB39B*, *RNF135*, *SETD2*, *TBC1D7*) was performed, by using the Ion AmpliSeq Library Kit Plus (Thermo Fisher Scientific, Waltham, MA, USA) for library preparation, following the manufacturer’s instructions, the Ion Chef instrument (Thermo Fisher Scientific, Waltham, MA, USA) for emulsion PCR, the Ion PGM (Thermo Fisher Scientific, Waltham, MA, USA) for sequencing, and the Ion Reporter software v.5.20 (Thermo Fisher Scientific, Waltham, MA, USA) for variant calling. ANNOVAR command-line software v. 2020Jun08 was used for variant annotation [9]. Variants classified as pathogenic or of uncertain significance were confirmed with Sanger sequencing and tested in both parents (when available). Segregation analysis was subsequently conducted by Sanger sequencing in parents, when available, for all the variants not already classified as benign after the initial annotation. We were able to test the segregation of the variants reported here in 20 out 22 patients, since the parents of 2 patients were not available for testing.

### 2.5. cDNA Analyses

Suspected splicing defects were evaluated by means of cDNA analyses. This analysis was assessed for the subject PTEN#13, carrier of NM_000314.8(*PTEN*):c.210-1G>C detected by HTS panel testing. Total RNA was obtained from short-term PHA-stimulated lymphocyte cultures established from patients’ heparinised blood samples, using the guanidinium thiocyanate–phenol–chloroform extraction method on both untreated and puromycin-treated cells, following standard procedures. cDNA was obtained by reverse transcription of RNA (High-Capacity cDNA Reverse Transcription Kit, Thermo Fisher Scientific, Waltham, MA, USA). We characterised potential splicing defects by cDNA sequencing. Primers were designed utilising the Primer3Input software tool (https://primer3.ut.ee/ (accessed on 1 February 2024)) and then validated with the UCSC In Silico PCR tool (https://genome.ucsc.edu/cgi-bin/hgPcr (accessed on 1 February 2024)) and the OligoCalc tool (http://biotools.nubic.northwestern.edu/OligoCalc.html (accessed on 1 February 2024)). Primers were synthesised on service by Merck KGaA/Sigma Aldrich (primers and experimental conditions are available upon request).

The skipping of exon 4 was detected on the cDNA of *PTEN* as a consequence of the NM_000314.8:c.210-1G>C variant. Since exon 4 consists of 44 nucleotides, a frameshift is introduced together with a premature stop codon.

## 3. Results

This section refers to the 22 patients with sequence variants in *PTEN* (NM_000314.8) (tot. 16), *PPP2R5D* (NM_006245.4) (tot. 4) and *NSD1* (NM_022455.4) (tot. 2).

Of the 78 patients included in this study, none had intragenic CNVs in *PTEN*, *NFIX*, or *NSD1*.

### 3.1. Genetic Findings

Gene variants were classified according to ACMG criteria [10], integrated by the “ClinGen PTEN Expert Panel Specifications to the ACMG/AMP Variant Interpretation Guidelines for PTEN Version 3.1.0” to classify variants in *PTEN* [11].

With respect to *PTEN* (NM_000314.8), 12/16 were defined as pathogenic variants (PVs) (c.697C>T, in 2 patients, c.144C>A, c.413A>G, c.517C>T, c.697C>T, c.368A>G, c.755A>G, c.510T>G, c.386delG, c.633C>G, c.464A>G), and 4/16 as likely pathogenic variants (LPVs) (c.623delG, in 2 patients, c.755A>T, c.210-1G>C, c.202T>C). The c.210-1G>C variant in the *PTEN* variant was classified as likely pathogenic based on the results of the cDNA analysis.

With respect to *PPP2R5D*, 3 variants were classified as pathogenic (c.592G>A, c.598G>A, and c.751G>A) and the remaining (c.599A>G) were classified as likely pathogenic.

Variants in *NSD1* (c.4928G>C and c.6164C>T) were classified as likely pathogenic (LPV).

### 3.2. Mode of Inheritance of Genetic Variants

Parental analysis was performed in 14/16 *PTEN* families and in the totality of the *PPP2R5D* and *NSD1* families.

*PTEN* variants were de novo on 7/14 occasions (50%) (including the patient whose clinical data are not included in this report), and were maternally (tot. 4) or paternally (tot. 3) inherited in the remaining 7 cases (50%).

*PPP2R5D* variants were de novo on 3/4 occasions (75%). Only one in four individuals (25%) inherited the variant from their apparently healthy mother.

### 3.3. Phenotypic Features

Consent for sharing clinical and molecular data was denied by the parents of one individual with a de novo pathogenic variant in *PTEN*. Therefore, detailed clinical information is provided for 21 individuals, as reported in Supplementary Tables S1–S3.

The clinical data of patients with PVs in *NSD1* are not extensively discussed, since their clinical presentation was fully consistent with Sotos syndrome.

Among individuals with PVs or LPVs in *PTEN*, 13/15 (87%) had a head circumference (HC) greater than +3 SD, whereas only one out four individuals with PVs in *PPP2R5D* (25%) exhibited a similar phenotype. More specifically, head circumference was between +4 SD and +7 SD in 11/15 patients (73%) with *PTEN* variants; it was between +2 SD and +2.5 SD in 3/4 (75%) patients with variants in *PPP2R5D*. Height was within normal limits, ranging from −1.5 SD to +1 SD in most individuals in both groups. However, 5/15 (33%) of *PTEN* individuals had a height between +1 SD and +2.5 SD.

Motor delay was unusual in both groups. Slight motor delay was recorded in 2/15 (13%) *PTEN* patients and in 1/4 (25%) *PPP2R5D* patients. Hypotonia was recorded in 3/4 (75%) *PPP2R5D* carriers and in 2/15 (13%) *PTEN* carriers. Speech delay was reported in 10/13 (77%) *PTEN* carriers aged > 3 years and in the totality of *PPP2R5D* carriers.

### 3.4. Developmental Delay, Intellectual Disability, and the Behavioural Phenotype

DD/ID was diagnosed in 11/14 (79%) *PTEN* carriers. Among them, ID was borderline to mild in 7/11 (64%), moderate in 2/11 (18%) and severe in 2/11 (18%) cases. All individuals with *PPP2R5D* PVs were diagnosed with DD/ID, which was borderline to mild in 3/4 (75%) and moderate in 1/4 (25%) cases. A total of three out of fourteen *PTEN* individuals had IQ scores within the normal range, although behavioural issues were recorded in each (Table S1).

Behavioural abnormalities were diagnosed in 11/12 (92%) *PTEN* carriers, with manifestations ranging from ASD, as observed in 6/11 (55%), to motor stereotypies, attention-deficit/hyperactivity/(ADHD) and repetitive behaviours in the remaining 5 (45%).

### 3.5. Seizure Disorder, EEG Anomalies, Neuroimaging Findings, and Comorbidities

Overt seizure episodes were never recorded.

Electroencephalographic (EEG) abnormalities were detected in 2/15 (13%) *PTEN* carriers and in 1/4 (25%) *PPP2R5D* carriers.

Neuroimaging abnormalities were observed in 13/14 (93%) individuals with *PTEN* PVs, consisting of nonspecific findings, including enlarged perivascular spaces and Chiari type I malformation.

Additional comorbidities were identified in 6/15 (47%) *PTEN* carriers, including, most commonly, thyroid nodules, particularly in individuals over the age of 9 years, of whom some underwent thyroidectomy, but also ectodermal abnormalities, fibromas, and lipomas. In the *PPP2R5D* cohort, only individual PPP2R5D#2 exhibited a unilateral resting hand tremor, which is currently under diagnostic evaluation (Table S2).

### 3.6. Craniofacial Features

No distinctive craniofacial features were observed in individuals with PVs in *PTEN*, whereas two out of four individuals with PVs in *PPP2R5D* exhibited some peculiar, although unspecific facial characteristics, including frontal bossing, short nose with anteverted nostrils, and slightly downturned mouth.

The clinical manifestations in both the cohorts of *PTEN* and *PPP2R5D* are summarised in Table 1.

## 4. Discussion

Neurodevelopmental disorders in children are highly heterogeneous, both clinically and genetically. In the face of the rapid growth of genomic variants being identified by the current high-throughput sequencing (HTS) methods, a crucial issue is the comprehension of pathomechanisms underlying these disorders, whether monogenic or multifactorial/oligogenic in nature. It can allow not only for the precise diagnosis in individual patients, but also for prognostic evaluation, for the assessment of the recurrent risk in families and, finally, for possible targeted therapies. Furthermore, the dramatic increase in the number of variants that need to be interpreted constitutes the main drawback of the use of HTS as first-tier genetic test in diagnostic settings, outside a deep pre-testing connection with the clinical phenotype. All these considerations are particularly appropriate for autism spectrum disorders (ASDs). Idiopathic ASDs, which account for the majority of cases of autism, have a multifactorial/oligogenic pathogenesis, and the recurrence risk in siblings is as high as 5–36% [12]. Additional clinical manifestations in idiopathic ASDs can include intellectual disability (ID), epilepsy, motor delay, and macrocephaly [13]. On the other hand, monogenic ASDs can present with very few additional signs, mimicking, to a large extent, the idiopathic counterpart.

We applied HTS of a small gene panel, including a total of 27 genes associated with overgrowth/macrocephaly to a highly selected series of 78 individual aged 1–19 years, who were referred because of ASD/behaviour abnormalities, associated with mild ID in most, and true or relative macrocephaly. Pathogenic variants in a single gene were detected in 22 cases (28%), affecting *PTEN* (16 subjects), *PPP2R5D* (4 subjects), and *NSD1* (2 subjects). In the two patients with the *NSD1* variant, the hypothesis of Sotos syndrome was in fact raised before the genetic test, clinically, based on the fully consistent phenotype of generalised overgrowth and the typical facial and hands characteristics. Patients with the *NSD1* variant are not discussed in this report. Thus, variants in *PTEN* and in *PPP2R5D* account for 25.6% of cases in our cohort (20/78).

Consent for reporting clinical information was denied by one *PTEN* family.

With respect to patients with variants in *PTEN* (tot. 15) and *PPP2R5D* (tot. 4), clinical manifestations were limited to macrocephaly and ASD/behavioural abnormalities, with borderline or mild ID in most cases and nonspecific additional features in a few cases. It should be noted that true facial dysmorphisms were never detected in either of the groups. Only minor facial anomalies, including frontal bossing, a short nose with anteverted nostrils, and a slightly downturned mouth, were detected in 3/4 patients with the *PPP2R5D* variant. It should also be noted that the neurodevelopmental impairment was very mild in the majority of patients in both groups.

Thus, *PTEN* and *PPP2R5D* patients could have all mimicked primary ASD at a first preliminary clinical evaluation.

Individuals with *PTEN*-related conditions in our cohort predominantly exhibited significant macrocephaly, height within normal limits, and subtle CNS anomalies, including perivascular space ectasia and Arnold Chiari 1 anomaly. It should be noted that similar CNS findings are described in several other patients described in the literature [14]. All the patients with *PTEN* variants had no peculiar facial characteristics. Hypotonia and motor delay were also unusual. Cognitive abilities were also relatively preserved, with most individuals falling within the borderline range or exhibiting only mild impairment. A small subset even had cognitive performance within the lower normal limits.

The severity of ASD symptoms varied widely among individuals. Some presented with attention-deficit/hyperactivity disorder (ADHD), stereotypies, anxiety, irritability, or self-injurious behaviours, while others had milder or more subtle behavioural issues.

However, the heterogeneity of neurocognitive and behavioural testing methods used, which is related to age and phenotype differences, limits a more detailed comparison of the patients in our cohort, with respect to their neurodevelopmental trajectories and behavioural profiles.

Based on our observations, and as confirmed by the data reported in the literature, the distinctive *PTEN*-associated phenotype in children appears to include true macrocephaly, more often above +4 SD, ASD or related clinical manifestations, borderline or mild ID, CNS anomalies limited to perivascular space ectasia, Arnold Chiari 1 anomaly, and no facial dysmorphisms. The constellation of these features could prompt clinicians to plan in silico analysis of the *PTEN* gene as a first-tier genetic test, and a search for partial gene deletion/duplication should also be planned in patients with an apparently normal status of *PTEN* on sequencing analysis [15].

Identifying *PTEN* variants has great clinical relevance also for cancer prevention and monitoring. In our cohort of patients aged 1–19 years, none had developed malignancies, as expected. There is in fact wide evidence that *PTEN*-associated malignant neoplasms are strongly age-related. However, three patients required total thyroidectomy in childhood due to multinodular goitre. Additionally, lipomas and fibromas were observed in some, reinforcing the need for ongoing surveillance. Importantly, about half of the variants in *PTEN* in our cohort were inherited, making the genetic diagnosis useful for the anticancer surveillance in families as well. It must be noted, however, that the heterogeneity of age in our patients may have limited the ascertainment of some phenotypic characteristics, including many *PTEN-*associated neoplasms, either benign or malignant in nature, which are not expected in childhood, and some neurodevelopmental and behavioural anomalies, such as speech delay and ADHD, which cannot be properly assessed in the very first years of life.

In contrast, the phenotype of individuals harbouring pathogenic variants in the *PPP2R5D* gene seems nonspecific. Following the first report of *PPP2R5D*-related neurodevelopmental disorder in patients who were described with mild to severe ID, hypotonia, susceptibility to epilepsy, facial dysmorphisms including frontal bossing, hypertelorism, and downslanting palpebral fissures, the number of reported cases has increased exponentially, thanks to the wide application of exome sequencing. Consequently, there has been a rise in case reports of *PPP2R5D* PVs carriers with a nonsyndromic clinical presentation [16].

Patients harbouring PVs in *PPP2R5D* exhibit a higher incidence of hypotonia, macrocephaly (relative more often than true), and language impairment, while a smaller percentage present with ASD and ID [17]. Although our cohort is limited to four patients, it reflects the nonspecific phenotype described in the literature, including the higher incidence of hypotonia in *PPP2R5D*-positive individuals compared to those with *PTEN* variants [18].

A final consideration is warranted. The aim of our report is not to define the diagnostic procedure for genetic testing in patients with intellectual disability or autism, which has already been discussed extensively in leading studies and guidelines proposed by authoritative groups [19]. Rather, this study deals with a centre-specific experience with neurodevelopmental disorders and behavioural abnormalities with a nonspecific clinical presentation, which at first glance could have mimicked a multifactorial/polygenic condition. In fact, the possibility of *PTEN* variants being causative for the observed phenotype was considered, clinically, in some of our patients, who were consequently enrolled into our HTS gene panel analysis as a first-tier genetic test, with positive results. All the remaining patients underwent array-CGH first, and then the HTS gene panel analysis. The frequency of *PTEN* mutations in our patient series is as high as 20.5%, one of the highest figures but still consistent with respect to data reported in the literature [19]: in fact, a great variability in the frequency of *PTEN* PVs in patients with ASD and macrocephaly has been reported, ranging from 1 to 22% [5], which most likely reflects the selection criteria of patients. It should be noted that mutations in *PPP2R5D* are relatively frequent in our cohort of patients as well, accounting for 5% of cases.

Guidelines have been proposed in relation to the application of exome sequencing (ES) or genome sequencing (WGS) in the diagnostic procedures of neurodevelopmental disorders, recommending their implementation as a first- or second-tier test in patients with a developmental or intellectual disability, while in isolated autism without ID or congenital malformation their application on a routine basis appears to be more controversial [20]. Nonetheless, it can depend on country-specific strategies, related to specific and diverse health policies. However, based on our results, a clinically oriented diagnosis can facilitate the extensive in silico analysis of restricted genes, including the search for intragenic copy number variants.

## 5. Conclusions

This study deals with a centre-specific approach to the diagnosis of the genetic causes of non-syndromic ASD and/or behavioural issues and macrocephaly, often associated with a variable degree of cognitive impairment. Together, these phenotypic features may be regarded as a clinical category for which the use of exome or genome sequencing as first-line testing is still a matter of debate [20] and may not be supported by certain healthcare systems due to their costs. Thus, a targeted approach may be more effective, especially if it includes the analysis of the *PTEN* gene, as per earlier guidelines [19], considering the consequences in terms of clinical management for *PTEN*-mutated patients. Though we report on a small cohort, and our hypotheses cannot be generalised, our findings are reinforced by their consistency with the previous literature, both considering the observed clinical manifestations and the frequency of *PTEN* PVs among patients with ASD and macrocephaly [19]. Nonetheless, our approach of HTS testing of a panel of genes constitutes an effective strategy for at least two further reasons, apart from the identification of *PTEN* variants:

(1) it is helpful in the diagnosis of specific syndromes in which macrocephaly and ASD/ID constitute a main feature, especially in those for which a milder presentation of sign and symptoms may hamper the clinical diagnosis;
(2) the inclusion of the *PPP2R5D* gene allows for the genetic diagnosis in a significant amount of patients with idiopathic macrocephaly and ASD, as our results would suggest.

## Figures and Tables

**Table 1:** Summary of clinical manifestations of individuals with PVs or LPVs in *PTEN PPP2R5D*.

*PTEN* and *PPP2R5D* Variants: Relevant Features	*PTEN* (Tot. 15) M/F: 10/5 Age (years): 1–19		*PPP2R5D* (Tot. 4) M/F: 2/2 Age (years): 2–16	
	N	%	N	%
**De novo**	6/13	46	3/4	75
**Paternally inherited**	3/13	23	0/4	0
**Maternally inherited**	4/13	31	1/4	25
**Macrocephaly (HC > +3 SD)**	13/15	87	1/4	25
**Macrocephaly (+2 SD < HC < +3 SD)**	2/15	13	3/4	75
**Height/Length (SD)**				
**−1.5 SD** **→** **+1 SD**	10/15	67	4/4	100
**+1 SD** **→** **+2.5 SD**	5/15	33	0/4	0
**Hypotonia**	2/15	13	3/4	75
**Motor delay**	2/15	13	1/4	25
**Speech delay**	10/13	77	4/4	100
**DD/ID**	11/14	79	4/4	100
**Borderline/mild**	7/11	64	3/4	75
**Moderate**	2/11	18	1/4	25
**Severe**	2/11	18	0/4	0
**ASD/Behavioural problems**	11/12	92	4/4	100
**Peculiar facial characteristics**	0/15	0	2/4 ^1^	50
**Epilepsy/EEG anomalies**	2/15 ^2^	13	1/4 ^2^	25
**CNS anomalies**	13/14 ^3^	93	0/4	0
**Comorbidities**	6/15 ^4^	40	1/4 ^5^	25

^1^ Frontal bossing (HP:0002007); Deeply set eye (HP:0000490); Thick vermilion border (HP:0012471); Short nose (HP:0003196) with anteverted nostrils (HP:0000463), Downturned mouth (HP:0002714). ^2^ EEG anomalies in absence of seizures. ^3^ Chiari I malformation: 5; Ectasia of the perivascular spaces: 5; Other: 3 (Table S1). ^4^ Multinodular goiter: 3; Lipomatosis: 1; Fibromas: 1; Ectodermal abnormalities: 3 (Table S1). ^5^ Unilateral resting hand tremor: 1 (Table S2).

## Data Availability

The data are contained within the article and the Supplementary Materials.

## Supplementary Materials

The following supporting information can be downloaded at: https://www.mdpi.com/article/10.3390/genes16040469/s1, Table S1: clinical and genomic data of *PTEN* subjects; Table S2: clinical and genomic data of *PPP2R5D* subjects; Table S3: clinical and genomic data of *NSD1* subjects.

## Abbreviations

The following abbreviations are used in this manuscript:

ID	Intellectual disability
ASD	Autism spectrum disorders
NDD	Neurodevelopmental disorder
HC	Head circumference
SD	Standard deviations
PV	Pathogenic variant
EEG	Electroencephalogram
HPO	Human Phenotype Ontology
HTS	High-throughput sequencing
ADHD	Attention-deficit/hyperactivity disorder
ES	Exome sequencing
WGS	Genome sequencing
tot	total
